# Glucose-Potentiated Amikacin Killing of Cefoperazone/Sulbactam Resistant *Pseudomonas aeruginosa*

**DOI:** 10.3389/fmicb.2021.800442

**Published:** 2022-03-03

**Authors:** Xi-kang Tang, Yu-bin Su, Hui-qing Ye, Zhen-yuan Dai, Huan Yi, Ke-xin Yang, Tian-tuo Zhang, Zhuang-gui Chen

**Affiliations:** ^1^Department of Pediatrics, The Third Affiliated Hospital, Sun Yat-sen University, Guangzhou, China; ^2^Department of Allergy, The Third Affiliated Hospital, Sun Yat-sen University, Guangzhou, China; ^3^Department of Cell Biology & Institute of Biomedicine, National Engineering Research Center of Genetic Medicine, MOE Key Laboratory of Tumor Molecular Biology, Guangdong Provincial Key Laboratory of Bioengineering Medicine, College of Life Science and Technology, Jinan University, Guangzhou, China; ^4^Department of Pulmonary & Critical Care Medicine, The Third Affiliated Hospital, Sun Yat-sen University, Guangzhou, China

**Keywords:** *Pseudomonas aeruginosa*, metabolomics, cefoperazone/sulbactam, glucose, amikacin, multidrug-resistant bacteria, the central carbon metabolism

## Abstract

Multidrug-resistant *Pseudomonas aeruginosa* has become one of global threat pathogens for human health due to insensitivity to antibiotics. Recently developed reprogramming metabolomics can identify biomarkers, and then, the biomarkers were used to revert the insensitivity and elevate antibiotic-mediated killing. Here, the methodology was used to study cefoperazone/sulbactam (SCF)-resistant *P. aeruginosa* (PA-R_SCF_) and identified reduced glycolysis and pyruvate cycle, a recent clarified cycle providing respiratory energy in bacteria, as the most key enriched pathways and the depressed glucose as one of the most crucial biomarkers. Further experiments showed that the depression of glucose was attributed to reduction of glucose transport. However, exogenous glucose reverted the reduction to elevate intracellular glucose *via* activating glucose transport. The elevated glucose fluxed to the glycolysis, pyruvate cycle, and electron transport chain to promote downstream proton motive force (PMF). Consistently, exogenous glucose did not promote SCF-mediated elimination but potentiated aminoglycosides-mediated killing since aminoglycosides uptake is PMF-dependent, where amikacin was the best one. The glucose-potentiated amikacin-mediated killing was effective to both lab-evolved PA-R_SCF_ and clinical multidrug-resistant *P. aeruginosa*. These results reveal the depressed glucose uptake causes the reduced intracellular glucose and expand the application of metabolome-reprogramming on selecting conventional antibiotics to achieve the best killing efficacy.

## Introduction

*Pseudomonas aeruginosa* is one of the most important opportunistic pathogens, whose clinical relevance is due to the high morbidity/mortality in subjects with compromised respiratory function, such as ventilator-associated pneumonia and cystic fibrosis. Besides, it can cause several acute and chronic infections, including infections of skin and soft tissues, meningitis, urinary tract, bone and joint, bacteremia corneal infections, abscess, conjunctival erythema, and a variety of systemic diseases associated with genetic diseases, also in immunocompromised patients, such as those with diabetes mellitus and individuals receiving chemotherapy (Al-Wrafy et al., 2017). Now, the bacterium is one of the top three causes of opportunistic human infections (De Simone et al., 2016).

Despite the significant advances in therapeutic practices, eradication of *P. aeruginosa* has become increasingly difficult (Pang et al., 2019). This is because the bacterium produces an armoury of products which modify its infective niche to ensure bacterial survival (Cripps et al., 1995). More importantly, strains of *P. aeruginosa* are known to utilize the high levels of intrinsic and acquired resistance mechanisms to deal with most antibiotics (Pang et al., 2019). However, antibiotics are still the first line of drugs to treat the infection caused by the bacterium.

In this background, multidrug-resistant *P. aeruginosa* strains are frequently isolated in clinic, which are resistant to at least three diverse classes of antibiotics including aminoglycosides, carbapenems, antipseudomonal penicillins, quinolones, and cephalosporins (Hirsch and Tam, 2010). In mainland China, a meta-analysis of 50 studies published from 2010 to 2014 is conducted for prevalence of antibiotic-resistant *P. aeruginosa* in patients with pneumonia including hospital-acquired pneumonia. The frequency of *P. aeruginosa* isolates resistant to agents recommended for the treatment of hospital-acquired pneumonia ranged 22.2% for amikacin to 50.0% for cefoperazone (Ding et al., 2016). Another investigation in emergency patients from 2013 to 2017 shows higher resistance rates of *P. aeruginosa* to cefoperazone/sulbactam (SCF) with the resistance increasing year over year, while the resistance rate to amikacin was below 10% (Huai et al., 2019). These investigations suggest that SCF-resistant *P. aeruginosa* is a great challenge to be controlled and amikacin can be a suitable drug for combatting multidrug-resistant *P. aeruginosa*. Therefore, the potentiation of amikacin-mediated killing efficacy is especially crucial in eliminating multidrug-resistant *P. aeruginosa* strains.

Recently developed metabolome-reprogramming is a powerful approach to combat antibiotic-resistant bacteria through enhancing the conventional antibiotics-mediated killing, even if the bacteria become insensitive to the antibiotics (Peng et al., 2015a,b; Zhang et al., 2019, 2020; Li et al., 2020; Wang et al., 2021). The approach uses comparative metabolome to identify crucial biomarkers between antibiotic-resistant and -sensitive metabolome and then utilizes the crucial biomarkers to reprogram the antibiotic-resistant metabolome and -sensitive metabolome, which makes bacteria become sensitive to the antibiotics. This approach has been demonstrated in several bacteria including *Escherichia coli*, *Edwardsiella tarda*, *Vibrio alginolyticus*, *P. aeruginosa*, and *Xanthomonas oryzae* (Peng et al., 2015a,b; Su et al., 2018; Cheng et al., 2019; Zhang et al., 2019, 2020; Li et al., 2020; Kuang et al., 2021). In these studies, an antibiotic is used to select antibiotic-resistant bacteria, and then, the same antibiotic with a crucial biomarker is utilized to kill the selected antibiotic-resistant bacteria, where the higher efficacy is found in the same class of drugs than other classes of drugs. For example, depressed alanine is identified from kanamycin-resistant metabolomes and exogenous alanine potentiates aminoglycosides (kanamycin and gentamicin) to kill kanamycin-resistant bacteria with higher efficacy than other classes of antibiotics including ampicillins (ampicillin), cephalosporins (ceftazidime), and quinolones (balofloxacin; Peng et al., 2015b). However, whether the crucial biomarker can potentiate the other classes of antibiotics with higher killing efficacy than the class of antibiotic used to select antibiotic-resistant bacteria is unknown.

Here, GC–MS based metabolomics approach was used to investigate differential metabolic profile between SCF-sensitive and -resistant *P. aeruginosa* ATCC27853 (PA-S and PA-R_SCF_, respectively). Glucose was depressed and identified as the most crucial biomarker. Exogenous glucose did not promote SCF-mediated killing but potentiate amikacin to kill lab-evolved PA-R_SCF_ and clinically evolved multidrug-resistant *P. aeruginosa*.

## Materials and Methods

### Bacterial Strains and Culture Conditions

ATCC 27853 and multidrug-resistant *P. aeruginosa* strains were from our lab stock. Overnight *P. aeruginosa* cultures were grown by inoculating cells from frozen stock into fresh LB. Cefoperazone/sulbactam (SCF)-resistant *P. aeruginosa* (PA-R_SCF_) was selected from ATCC 27853 sequentially generating in LB medium (1% bacterial peptone, 0.5% yeast extract, and 1% NaCl) plus 1/2 MIC of SCF (4 μg/ml). At the same time, ATCC 27873 was sequentially generated in LB medium without antibiotic as control SCF-sensitive *P. aeruginosa* (PA-S). The sequential generations led to SCF-resistant *P. aeruginosa* with 8MIC (PA-R8_SCF_) and control PA-S8 at 9 days/generations and with 16 MIC (PA-R16_SCF_) and control PA-S16 at 24 days/generations.

### Measurement of MIC

Bacteria for MIC measurement were obtained by re-inoculating the overnight cultures at 1:100 dilutions into 50 ml LB and grown until OD_600_ 0.5. SCF was diluted by serial 2-fold dilution steps ranging from 0.25 to 256 μg/ml in LB medium and transferred into the 96 well plates. Bacterial cells with 10^5^ colony-forming units (CFU) were added and incubated at 37°C for 16 h. The lowest concentration which inhibited the visual growth was recorded as MIC. Data were obtained from three biological replicates.

### Survival Capability

Survival capability test was performed as previously described (Li et al., 2007). Frozen stocks were grown overnight on fresh LB at 37°C. The saturated bacteria were diluted (1:1,000) into fresh LB medium and added the corresponding antibiotics. The final antibiotic concentrations of 1/16MIC, 1/8MIC, 1/4MIC, 1/2MIC, 1MIC, 2MIC, 4MIC, 8MIC, and 16MIC were set with the measured initial strain MIC as the antibiotic concentration scale. After shaking at 37°C for 6 h, the OD_600_ was measured, and survival rate was calculated in each group and without antibiotics was used as control.

### Growth Curve Measurement

The overnight bacteria were diluted 1:100 in LB medium at 37°C with 200 rpm and OD_600_ values were measured at 0, 2, 4, 6, 8, 10, and 12 h. The growth curve under these conditions was drawn by plotting the OD values and the corresponding culture time.

### Effect of Sugar on Growth

Overnight bacterial cultures were collected by centrifugation at 8,000 rpm for 5 min. The samples were washed three times with sterile saline and then resuspended in M9 minimal medium containing 0.1 g/L MgSO_4_ and 0.588 g/L sodium citrate (Meylan et al., 2017). The resuspension was diluted to 0.2 of OD_600_. Glucose, gluconate, 2-ketogluconate, glucose-6-phosphate, fructose, galactose, maltose, or mannose was added to a final concentration of 5 mm and incubated at 37°C and 200 rpm for 4 h. Finally, OD values were determined to compare the effect of the carbon source on the growth of antibiotic-sensitive and -resistant *P. aeruginosa*.

### GC–MS Analysis

GC–MS analysis was performed as previously described (Cheng et al., 2019). In brief, samples for GC–MS analysis were prepared by re-inoculating these cultures at 1:100 dilutions into 50 ml LB and incubated for 5 h until OD_600_ 1.0. Equivalent numbers of cells were quenched with −80°C pre-cooled methanol (Sigma) and then centrifuged at 8,000 rpm at 4°C for 5 min, followed by extraction of metabolites using 1 ml of cold methanol. To normalize variations across samples, an internal standard (0.1 mg ml^−1^ ribitol; Sigma) was used. The cells were treated with supersonic waves for 10 min at 4°C and then centrifuged at 12,000 rpm at 4°C for 10 min. Supernatant with 800 μl was transferred into a new microcentrifuge tube. Extracts using for GC–MS analysis were dried in vacuum centrifuge and 80 μl of 20 mg/ml methoxyamine hydrochloride (Sigma) in pyridine was added for 180 min at 37°C. Then, 80 μl N-methyl-N-(trimethylsilyl) trifluoroacetamide (MSTFA, Sigma) was added for derivatization for 30 min at 37°C. The derivatized sample with 1 μl was injected to DBS-MS column. Initial temperature was 85°C for 5 min, followed by an increase to 270°C at a rate of 15°C/min and held for 5 min. Helium was used as carrier gas at constant flow with a rate of 1 ml/min. The MS scan range was at 50–600 *m/z*. The GC–MS data were detected with an Agilent 7890A GC equipped with an Agilent 5975C VL MSD detector (Agilent Technologies). Four biological repeats with two technical replicas were performed each strain.

Initial peak detection and mass spectral deconvolution were carried out with Agilent software (Agilent 6.0). Metabolites were identified according to spectral matching and retention time (RT) by searching in the National Institute of Standards and Technology (NIST) Mass Spectral Library. Data matrix was normalized using internal standard (ribitol) and the total intensity. Software IBM SPSS Statistics 22 was used to analyze statistical difference, and value of *p* < 0.01 was considered significant. Hierarchical clustering was completed in the R platform with the package gplots^1^ using the distance matrix. *Z*-score was used to analyze normalized area of differential metabolites. Multivariate statistical analysis included principal component analysis (PCA) and S-plot analysis were performed by SIMCA-P + 12.0.1 software (Umetrics, Umea, Sweden). Enrichment of significant metabolic pathways was conducted with MetaboAnalyst 5.0. GraphPad Prism 8.0 was used to draw figures.

### Antibiotic Bactericidal Assay

Antibacterial assay was carried out as previously described with a modification (Meylan et al., 2017; Kuang et al., 2021). Stationary-phase cells were prepared by growing cells in LB medium until OD_600_ 0.2 and then diluted at 1:1,000 to 50 ml LB and cultured for 16 h. The bacterial cells were harvested by centrifugation and suspended in M9 medium to 0.2 of OD_600_ nm. Desired concentrations of antibiotics and glucose were added and then incubated at 37°C, 200 rpm for 6 h. To determine bacterial counts, 100 μl of cultures were acquired and serially diluted. Aliquot of 5 μl of each dilution was spotted in LB agar plates and cultured at 37°C for 18 h. The plates only yielding 20–200 colonies were counted and CFU/ml was calculated. Bio-repeats were run in triplicate.

### Measurement of Intracellular Glucose Concentration

The bacterial cells used for glucose concentration measurement were the same as for antibacterial assay. Glucose assay kit (Solarbio BC2505) was used to assess glucose concentration in bacterial cells. Briefly, the cells were broken *via* ultrasonic disruption (35%, 2 s, 3 s interval, 8 min) at 4°C, boiled at 100°C for 10 min. The supernatant was acquired by centrifugation at 8,000 *g* for 10 min. Samples, standard solution, and reagent were added to a 96-well plate depending on manufacturer’s instructions and incubated at 37°C for 15 min. Absorbance was read at 505 nm. Experiments were performed at least three biological replicates.

### Quantitative Real-Time PCR

The bacterial cells used for quantitative real-time PCR (qRT-PCR) were the same as for antibacterial assay. Quantification of gene expression was performed as previously described. Briefly, total RNA was isolated from bacterial samples by TRIzol reagent (Ambion). cDNA was prepared with Evo M-MLV RT Kit with gDNA Clean for qPCR II (Accurate Biotechnology, Hunan, China). qRT-PCR was conducted in 384-well plates with SYBR Green Premix Pro Taq HS qPCR Kit (Accurate Biotechnology, Hunan, China) as directed by the manufacturer. Cycling parameters were 95°C for 30 s and 40 cycles of 95°C for 5 s; and 60°C for 30 s. Each sample were done with triples, and the data were normalized to reference genes 16S rRNA as previously described (Kuang et al., 2021), gene expression was calculated according to the 2^−ΔΔCT^ method. All the primers were listed in (Supplementary Table 1).

### Determination of NADH

NADH was measured with the EnzyChrom™ NAD^+^/NADH assay kit (BioAssay Systems) according to the manufacturer’s instructions. In brief, the bacterial cells used for NADH determination were the same as for antibacterial assay. Bacterial cells were harvested and washed with sterile saline three times. The cells were centrifuged for 3 min at 12,000 rpm. The resulting cells were resuspended in NADH extraction buffer and vortex for 15 s and incubated in 60°C water both for 5 min. Assay buffer and an equal volume of opposite extraction buffer (NAD) immediately. After vortex briefly and centrifugation at 14,000 rpm for 5 min, the supernatant sample was used for measurement and standard curve determination according to the manufacturer’s instructions.

### Membrane Potential Measurement

Membrane potential was measured by BacLight Bacterial Membrane Potential Kit (Invitrogen). The bacterial cells used for membrane potential measurement were the same as for antibacterial assay. The bacteria were diluted to 10^6^ CFU/ml with 10 μl of 3 mm DiOC_2_ (3) for 30 min with shaking at 37°C under dark conditions. The samples were analyzed by FACSCalibur flow cytometer (Becton Dickinson, San Jose, CA, United States) using the red light fluorescence intensity (Y mean) and green light fluorescence intensity (X mean). The membrane potential was measured and normalized to the intensity ratio of red fluorescence and green fluorescence.

### Measurement of ATP

ATP content was determined by BacTiter-Glo™ Microbial Cell Viability Assay (Promega). The bacterial cells used for ATP measurement were the same as for antibacterial assay. Samples with 50 μl were added to 96-well plate and mixed with same volume of kit solution. After reacting for 5 min, the fluorescence value was detected and the intracellular ATP content was calculated according to the standard curve.

### Enzyme Activity Determination

Measurement of enzyme activity was performed as previously described (Jiang et al., 2020a; Su et al., 2021). Briefly, bacterial cells were suspended in 1× PBS (pH 7.0) and adjust the OD_600_ to 1.0. Aliquot of 30 ml of cells were centrifuged and transferred to a 1.5 ml centrifuge tube. The cells were resuspended with 1 ml of 1× PBS and sonic oscillation for 6 min (total power 200 W, 35% output, pulse for 2 s, pause for 3 s) on the ice. Following by centrifugation at 12,000 rpm for 10 min at 4°C, supernatants were harvested. The supernatant protein concentration was determined by BCA protein concentration determination kit (Beyotime). Then, 300 μg proteins were used to determine the enzyme activity. For the measurement of PDH and KGDH, the reaction mixture included 0.15 mm 3-(4,5-dimethyl-2-thiazolyl)-2,5-diphenyl-2H-tetrazolium bromide (MTT), 2.5 mm MgCl_2_, 6.5 mm phenazine methosulfate (PMS), 0.2 mm thiamine PPi (TPP), 80 mm sodium pyruvate/α-ketoglutarate potassium salt, adding distilled deionized water with a final volume of 200 μl in 96-well plate. For SDH and MDH measurement, the reaction mixture included 0.5 mm MTT, 2.5 mm MgCl_2_, 130 mm PMS, 80 mm sodium succinate/sodium malate, adding distilled deionized water to 200 μl. All reactions were incubated at 37°C for 5 min for PDH/KGDH/MDH, except SDH was incubated for 10 min. Finally, absorbance at 562 nm was measured. Hexokinase (HK) and pyruvate kinase (PK) activity were measured with the HK or PK Activity Assay Kit (Boxbio Science & Technology, China) respectively. Cytochrome c oxidase (CcO) was assayed by the kit for bacteria (Genmed Scientifics Inc., United States). According to the manufacturer’s instructions, supernatants of ultrasonic disruption were carefully prepared and the protein concentration was measured. Aliquot of 300 μg proteins were took for determination uniformly. Activity of HK and PK was read at 340 nm, while activity of CcO was measured at absorbance of 550 nm. All reactions were protected from light. All assays were performed in three biological replicates.

### Detection of Amikacin Concentration

Intracellular amikacin was detected by ELISA rapid diagnostic kit (Mei Mian Biotechnology Co., Ltd., China), according to the kit instructions. The overnight bacteria were suspended to OD_600_ of 0.2 in M9 medium with or without 5 mm glucose plus amikacin in a shaker bath at 37°C for 4 h. The cells were washed and resuspended in sterile saline and adjusted to OD_600_ of 1.0. Each aliquot of 1 ml sample was sonicated for 3 min and centrifuged to remove insoluble matters. The resulting supernatant was collected for the detection of amikacin following the instructions of the kit. The optical density was determined by microplate reader at 450 nm.

### Statistical Analysis

Software IBM SPSS Statistics 22 was used for statistical analysis. Statistical difference was obtained by two-sided Mann–Whitney *U* test or *t* test. Value of *p* < 0.05 was considered significant. For the GC–MS analysis, four biological repeats with two technical replicas were performed, and the other experiments were performed at least three biological replicates.

## Results

### Phenotypes of PA-R_SCF_

**Figure 1:**
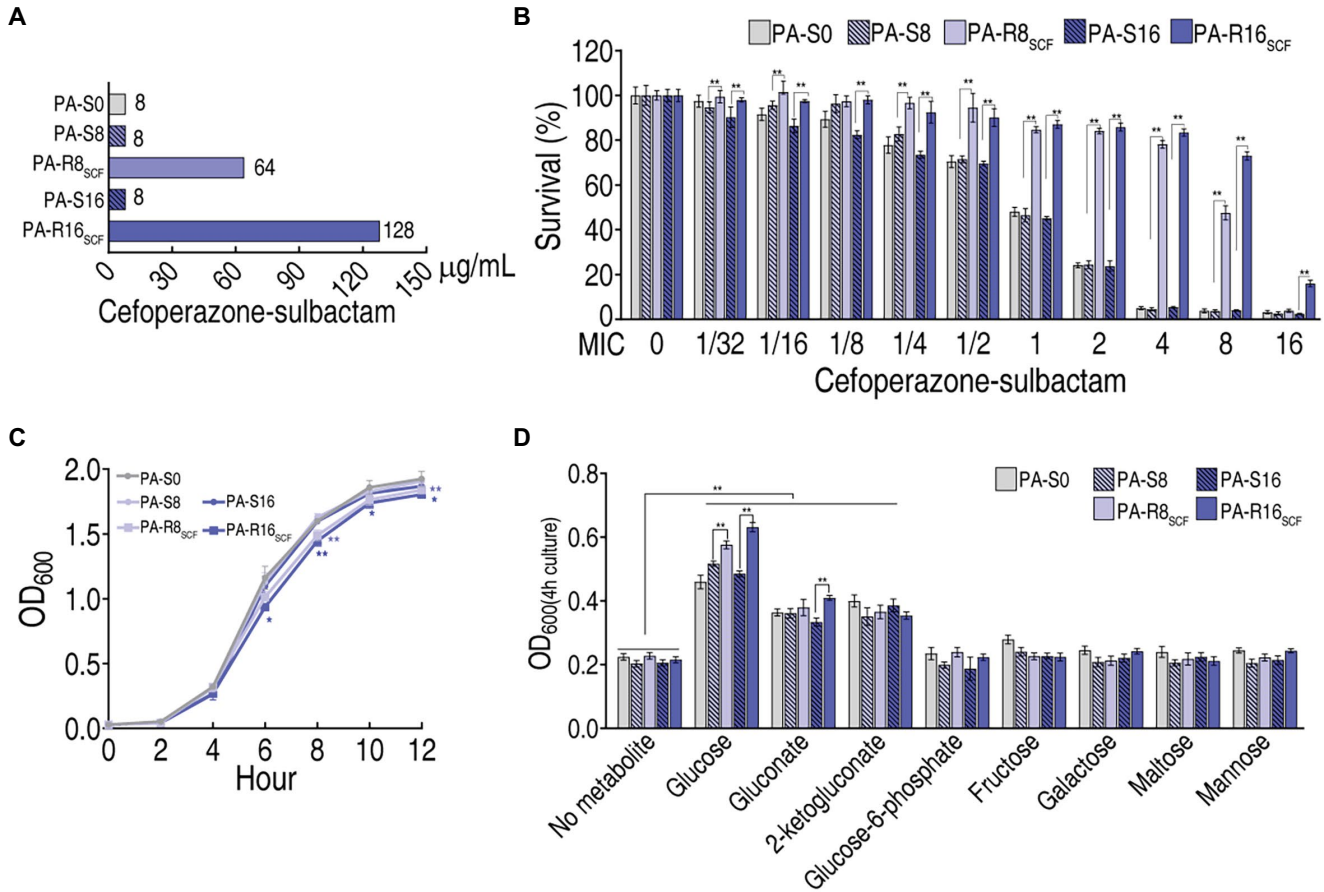
Resistance to cefoperazone/sulbactam (SCF) and effect of sugar on growth in PA-R_SCF_. **(A)** Minimum inhibitory concentration (MIC) of three PA -S and two PA -R strains. **(B)** Survival capability of three PA-S and two PA-R strains. **(C)** Growth curve of three PA-S and two PA-R strains. **(D)** Effect of the indicated sugars on growth of three PA-S and two PA-R strains. Results are displayed as mean ± SEM and three biological repeats are performed. Significant differences are identified ^*^p < 0.05, ^**^p < 0.01.

ATCC 27853 (PA-S0) was cultured in LB medium with or without SCF and evolved to PA-R8_SCF_ and PA-R16_SCF_ and became corresponding controls PA-S8 and PA-S16, respectively. The three PA-S strains had the same minimum inhibitory concentration (MIC) to SCF, which was defined 1MIC (8 μg), when PA-R8_SCF_ and PA-R16_SCF_ had 8 (64 μg) and 16 (128 μg) MIC, respectively (Figure 1A). Survival capability of the five strains ranged from high to low was PA-R16_SCF_ > PA-R8_SCF_ > three PA-S strains in SCF dose-dependent manner (Figure 1B), while lower growth was detected in the two PA-R strains than the three PA-S strains (Figure 1C). Among eight sugars detected, glucose, gluconate and 2-ketogluconate promoted growth of the five strains, where higher growth was measured in supplement of glucose in a MIC-dependent manner and of gluconate in PA-R16_SCF_ than PA-S16. However, glucose-6-phosphate, fructose, galactose, maltose, and mannose did not elevate the growth of the five strains (Figure 1D). These results that glucose-related metabolism is changed in the two PA-R strains.

### Metabolic Profiles of PA-R_SCF_

**Figure 2:**
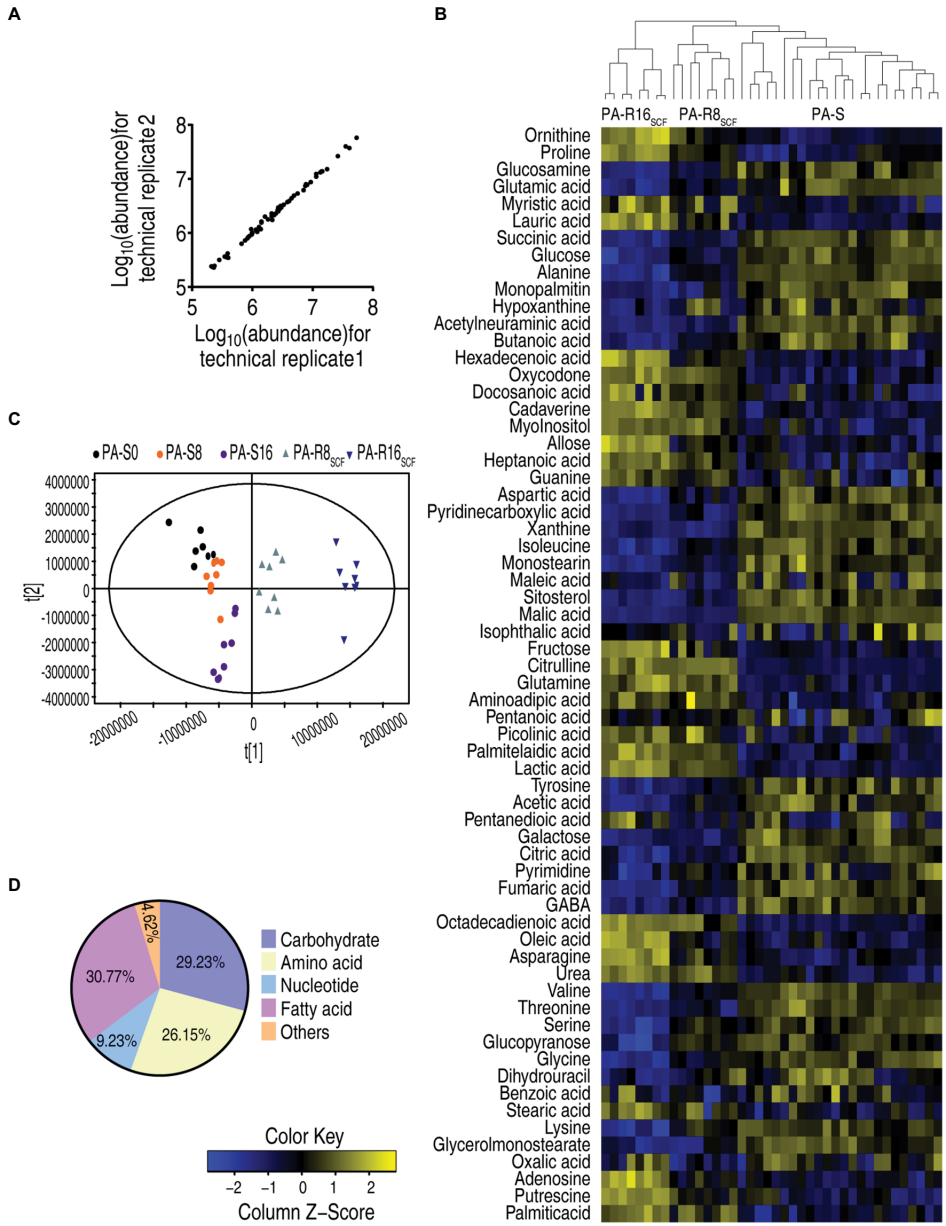
Metabolic profiles between three PA-S and two PA-R strains. **(A)** Reproducibility of the metabolomic profiling platform used in the discovery phase. Abundance of metabolites quantified in samples over two technical replicates is shown. The Pearson correlation coefficient between technical replicates varies between 0.998 and 0.999. **(B)** Heat map of unsupervised hierarchical clustering of different metabolites (row). Blue and yellow indicate decrease and increase of the metabolites scaled to mean and SD of row metabolite level, respectively (see color scale). **(C)** Principal component analysis (PCA) of the three PA-S and two PA-R strains. Each dot represents the technical replicate of samples in the plot. **(D)** Categories of the metabolites. Sixty-four metabolites are searched against in KEGG for their categories, and the pie chart is generated in Excel 2010 (Microsoft, United States).

The above results suggest that metabolism was affected in the two PA-R strains. Therefore, GC–MS-based metabolomics approach was used to investigate metabolic profiles among the five strains cultured in medium without SCF. Four biological samples with two technical replicates were performed in each strain, yielding 40 data sets. After the removal of internal standard ribitol and any known artificial peaks and the integration of the same compounds, 64 metabolites with reliable signals were identified in each strain. The correlation coefficient between technical replicates varied between 0.998 and 0.999 (Figure 2A), demonstrating the reproducibility of the data. Metabolic profiles of the five strains were displayed as a heatmap, where three PA-S and two PA-R were separated clearly and PA-R8_SCF_ and PA-R16_SCF_ were further sub-clustered, while three PA-S strains were mixed together (Figure 2B). Orthogonal partial least square discriminant analysis (OPLS-DA) was used to recognize the sample pattern. Component t [1] identified the two PA-R strains from the three PA-S strains, and component t [2] differentiated variants within the five strains. Notably, PA-R8_SCF_ and PA-R16_SCF_ were separately located each other; PA-S0 and PA-S16 were also separated, but PA-S8 bridged them (Figure 2C). Based on KEGG^2^ annotation and NCBI PubChem,^3^ the metabolites were classified into five categories, carbohydrates (29.23%), amino acids (26.15%), nucleotides (9.23%), fatty acids (30.77%), and others (4.62%; Figure 2D). These results indicate that SCF induces metabolic profile shifts in these sequentially generated strains.

### Differential Metabolome of PA-R_SCF_

**Figure 3:**
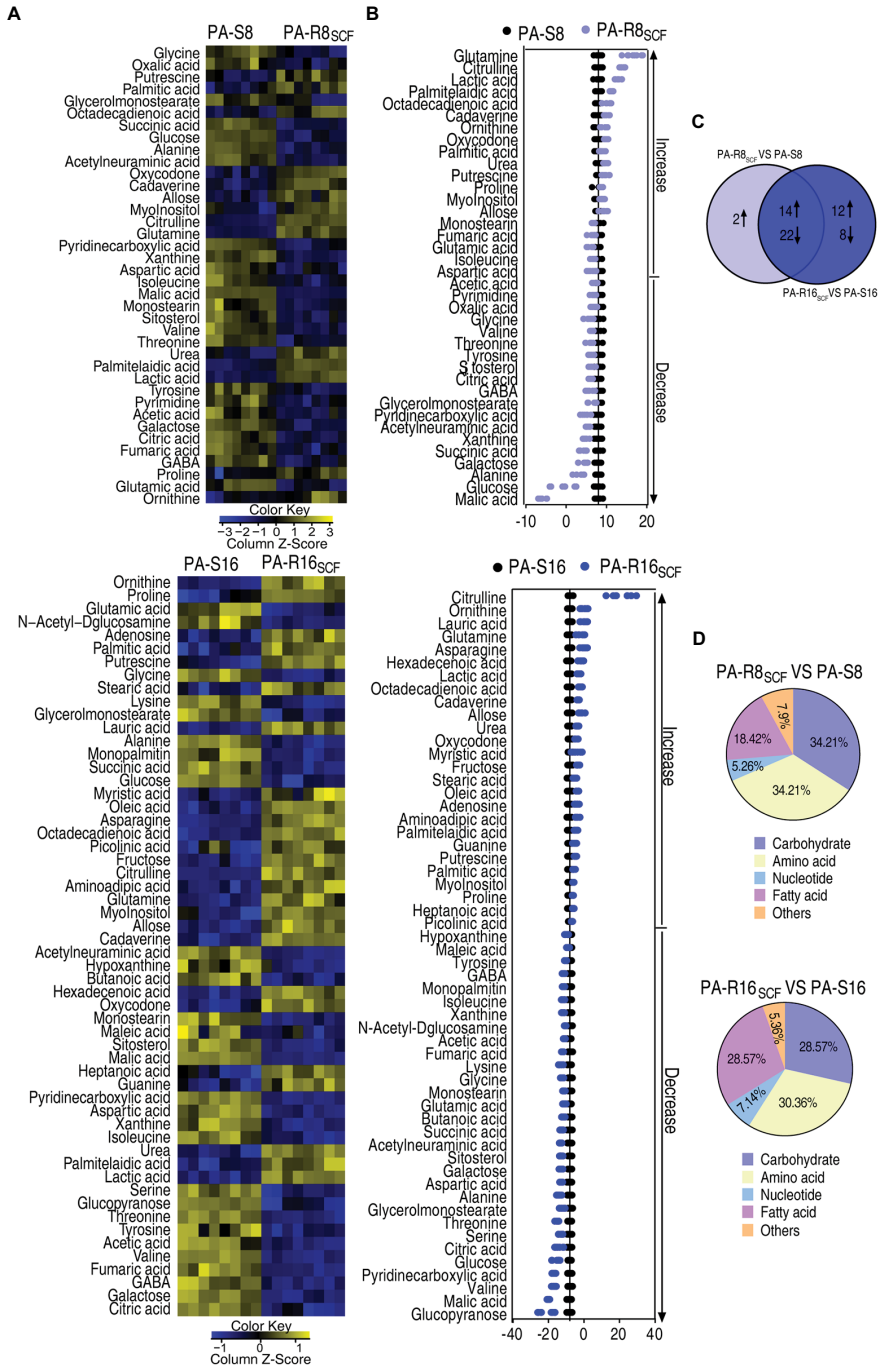
Differential metabolome of SCF-resistant *Pseudomonas aeruginosa*. **(A)** Heat maps showing differential metabolites in PA-R8_SCF_ and PA-R16_SCF_ compared with those in PA-S8 and PA-S16, respectively. Yellow color and blue color indicate increase and decrease of metabolites relative to the median metabolite level, respectively (see color scale). **(B)** Z-score plot of differential metabolites in PA-R8_SCF_ and PA-R16_SCF_ based on controls PA-S8 and PA-S16, respectively. The data were separately scaled to the mean and SD of the controls. Each point represents one metabolite in one technical repeat and colored by sample types. **(C)** Venn diagram for comparison of differential metabolites between PA-R8_SCF_ and PA-R16_SCF_. **(D)** Categories of the differential metabolites. Differential abundances of metabolites are searched against in KEGG for their categories, and the pie chart is generated in Excel 2010 (Microsoft, United States).

Then, a two-sided Mann–Whitney *U* test coupled with a permutation test was used to identify differential abundances of metabolites among the five strains. Out of the 64 metabolites, 38 and 56 with differential abundances were determined in PA-R8_SCF_ and PA-R16_SCF_, respectively, compared with PA-S8 and PA-S16 (*p* < 0.01). They were displayed as two heatmaps (Figure 3A). Z-score exhibited metabolite variation, with 16 increase and 22 decrease in PA-R8_SCF_ and 26 increase and 30 decrease in PA-R16_SCF_ (Figure 3B). Among the 38 and 56 differential abundances of metabolites, 14 upregulation and 22 downregulation were overlapped between PA-R8_SCF_ and PA-R16_SCF_ (Figure 3C). According to KEGG annotation and NCBI PubChem, the differential abundances of metabolites in PA-R8_SCF_ and PA-R16_SCF_ were classified into five categories, carbohydrates (34.21 and 28.57%), amino acids (34.21 and 30.36%), nucleotides (5.26 and 7.14%), fatty acids (18.42 and 28.57%), and others (7.90 and 5.36%), respectively (Figure 3D). These results indicate that most of differential abundances of metabolites are overlapped between PA-R8_SCF_ and PA-R16_SCF_. Meanwhile, more changes are detected in PA-R16_SCF_ than PA-R8_SCF_.

### Differential Metabolic Pathways of PA-R_SCF_

To understand which pathways the differential abundances of metabolites belong to, they were analyzed and outlined in KEGG (release 41.1)^4^ and MetPA.^5^ Total 10 metabolic pathways were enriched in both PA-R8_SCF_ and PA-R16_SCF_, where nine were overlapped and unique methane metabolism and lysine degradation were determined in PA-R8_SCF_ PA-R16_SCF_, respectively (Supplementary Figure 1A). The five most impacted pathways were alanine, aspartate and glutamate metabolism; arginine biosynthesis; TCA cycle; arginine and proline metabolism; and pyruvate metabolism (Supplementary Figure 1A). Out of the five metabolic pathways, TCA cycle showed all metabolites detected were reduced, while alanine, aspartate, and glutamate metabolism; pyruvate metabolism displayed most metabolites detected was decreased. Besides, all of four metabolites detected were also decreased in butanoate metabolism, the 8th impacted pathway, but three out of the four metabolites were overlapped with metabolites in the TCA cycle (Supplementary Figure 1B). The TCA cycle and pyruvate metabolism are located in the central carbon metabolism and alanine, aspartate, and glutamate metabolism fuels the TCA cycle. A recent report has indicated that the pyruvate cycle (the P cycle) rather than the TCA cycle provides respiratory energy in bacteria (Su et al., 2018). Therefore, inactivation of the P cycle is a characteristic feature in PA-R strains.

### Crucial Biomarkers of PA-R_SCF_

**Figure 4:**
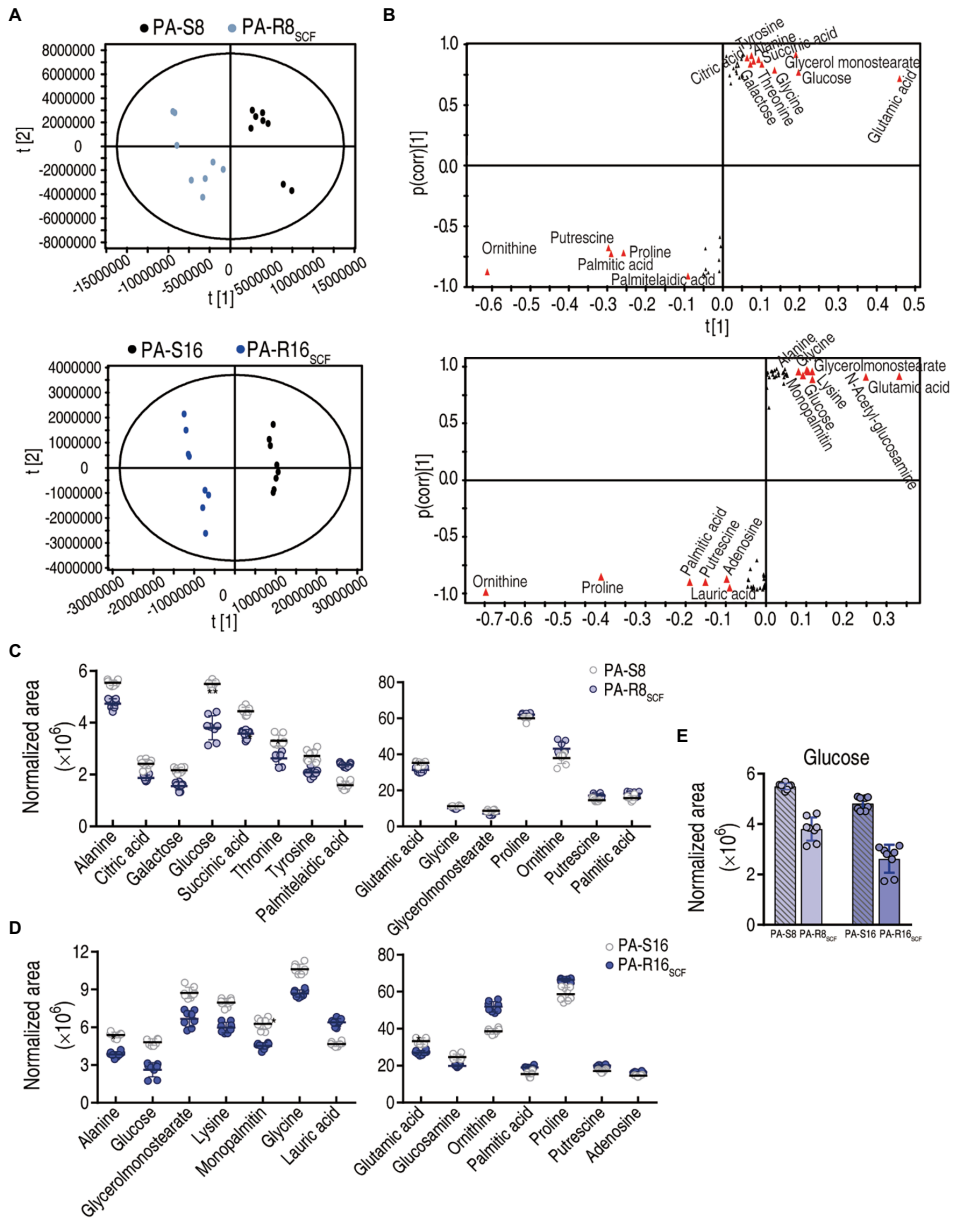
Identification of crucial metabolites. **(A)** The PCA analysis between PA-R8_SCF_, PA-R16_SCF_, and PA-S8, PA-S16, respectively. Each dot represents the technique replicates in the plot. **(B)** S-plot generated from OPLS-DA. Triangle represents individual metabolite, where potential biomarkers are highlighted with red, which is greater or equal to 0.05 and 0.5 for absolute value of covariance p and correlation p(corr), respectively. **(C–E)** The scatter plot of biomarkers in data **(B)**.

To explore the most crucial metabolites differentiating PA-S and PA-R, orthogonal partial least square discriminant analysis (OPLS-DA) was conducted to recognize the sample pattern. Component p [1] separated the two PA-R strains from the corresponding controls, and component p [2] differentiated variants within the four strains (Figure 4A). Further S-plot analysis was used to show discriminating variables, where cutoff values were set as greater or equal to 0.05 and 0.5 for the absolute value of covariance p and correlation p(corr), respectively. Totally, 15 and 14 biomarkers were identified in PA-R8_SCF_ and PA-R16_SCF_, respectively. Among them, the reduced glutamic acid, glucose, glycine, alanine, glycerol monostearate and elevated proline, ornithine, putrescine, and palmitic acid were overlapped between PA-R8_SCF_ and PA-R16_SCF_, whereas the decreased succinic acid threonine, galactose, citric acid, tyrosine and increased palmitelaidic acid were detected only in PA-R8_SCF_ and the decreased lysine, glucosamine, monopalmitin and increased lauric acid, adenosine only in PA-R16_SCF_ (Figure 4B). Differential abundance of these metabolites is shown in (Figures 4C–E). These results indicate that the central carbon metabolism is inactivated, where glucose serving for the fuel of origin is reduced and thereby plays a crucial role in the resistance.

### Disorder of Glucose Uptake and Phosphorylation and Reverting in PA-R16_SCF_

**Figure 5:**
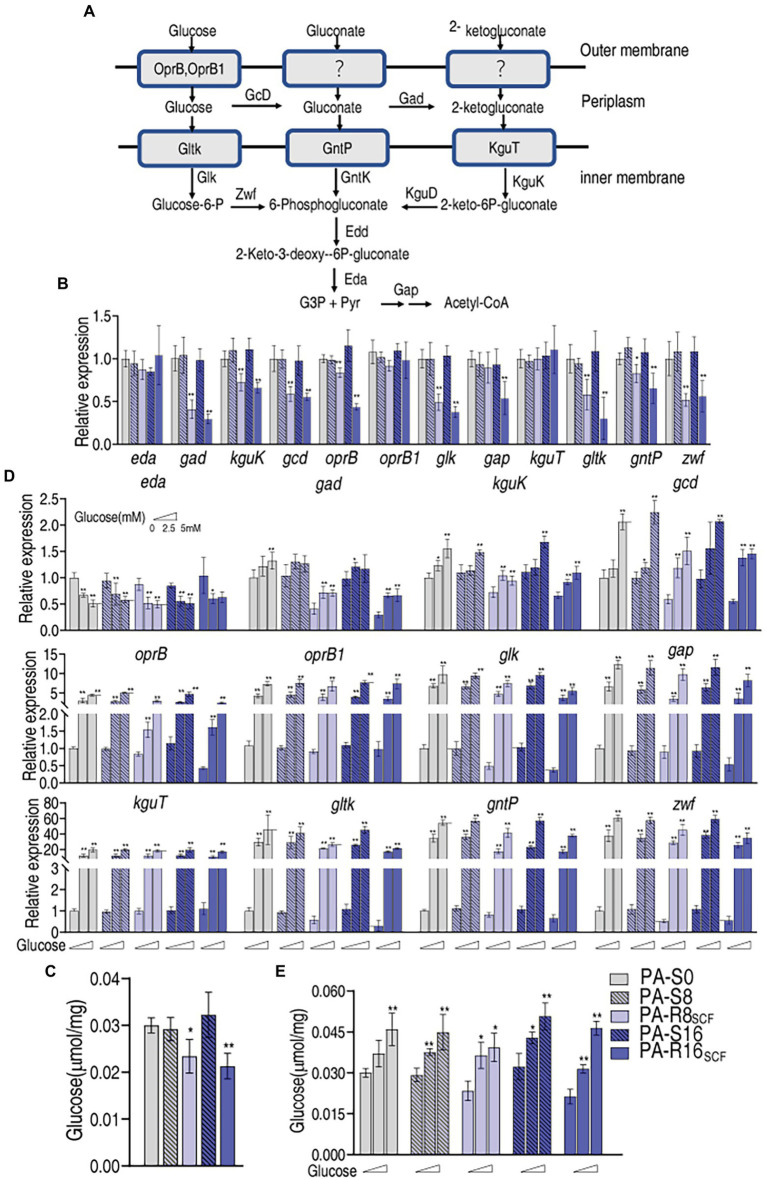
Glucose transport with and without exogenous glucose in PA-R8_SCF_ and PA-R16_SCF_. **(A)** Metabolic diagram of glucose transport (Daddaoua et al., 2017). **(B)** Quantitative real-time PCR (qRT-PCR) for expression of genes encoding glucose transport proteins between PA-R8_SCF_, PA-R16_SCF_, and PA-S8, PA-S16, respectively. **(C)** Glucose level in data **(B)**. **(D)** qRT-PCR for expression of genes encoding glucose transport proteins between PA-R8_SCF_, PA-R16_SCF_, and PA-S8, PA-S16, respectively, in the presence of different concentrations of glucose. **(E)** Glucose level in data **(D)**. Results are displayed as mean ± SEM and at least three biological repeats are performed. Significant differences are identified ^*^p < 0.05, ^**^p < 0.01.

Intracellular glucose concentration is dependent on uptake and utilization. A line of evidence has indicated that the central carbon metabolism fluxed by glucose is affected in antibiotic-stressed and -resistant bacteria (Peng et al., 2015b; Zhang et al., 2020). However, information regarding glucose transport including uptake and phosphorylation is not available. We supposed that the depressed glucose concentration is related to glucose uptake and phosphorylation. To test this idea, qRT-PCR was used to measure expression of genes encoding glucose uptake and phosphorylation. In the genus *Pseudomonas*, glucose metabolism occurs exclusively through the Entner–Doudoroff pathway with a two-step pathway comprising the 6-phosphogluconate dehydratase (Edd) and the 2-keto-3-deoxy gluconate aldolase (Eda) that convert 6-phosphogluconate into glyceraldehyde-3-phosphate and pyruvate (Figure 5A). A three-pronged metabolic system generates 6-phosphogluconate from glucose. Glucose is transported into periplasm by *oprB* and PA2291 encoding OprB and OprB1. Within the periplasm, glucose is converted into gluconate by GcD and then 2-ketogluconate by Gad. GltK, GntP, and KguT transporters mediate the uptake of these three compounds into the cytosol and then phosphorylated by GlK, GntK, and KguK, respectively. The resulting glucose-6-P and 2-keto-6P-gluconate are transformed to 6-phosphogluconate by Zwf and KguD, respectively (Figure 5A; Daddaoua et al., 2017). Therefore, expression of 12 genes encoding these proteins was measured. Lower expression of *gad*, *kguK*, *gcd*, *oprB*, *glk*, *gltK*, *gntP*, and *zwf* was found in PA-R16_SCF_ and PA-R8_SCF_ than PA-S16 and PA-S8, respectively, and exhibited a MIC-dependent manner in almost genes except for *zwf*, which was similar between PA-R16_SCF_ and PA-R8_SCF_. In addition, lower expression of *gap* was detected only in PA-R16_SCF_. However, no difference in expression of *eda*, *oprB1*, and *kguT* was determined between PA-R8_SCF_, PA-R16_SCF_, and PA-S8, PA-S16, respectively (Figure 5B). Consistently, intracellular glucose was lower in PA-R16_SCF_ and PA-R8_SCF_ than the three PA-S strains and displayed a MIC-dependent manner (Figure 5C). These results indicate that glucose transport is reduced in antibiotic-resistant *P. aeruginosa*, which is an important reason leading the depressed glucose.

It is known that extracellular glucose serves as OprB and OprB2 substrate, while GlK, GntK, and KguK phosphorylate periplasm glucose, gluconate, and 2-ketogluconate, respectively. Exogenous glucose must promote expression of the 12 genes of glucose transport. To demonstrate this, qRT-PCR showed that expression of 11 genes was elevated with increasing extracellular glucose concentrations except for *eda*, which was lower in medium with than without glucose (Figure 5D). Consistently, glucose concentrations were increased in an exogenous glucose dose-dependent manner (Figure 5E). These results indicate that expression of genes encoding glucose transport is reduced in PA-R compared with PA-S, which is related to MIC and responsible for the depressed glucose concentration in PA-R_SCF_. However, the reduction can be reverted by increasing extracellular glucose.

### Elevated Sensitivity to Amikacin by Exogenous Glucose

**Figure 6:**
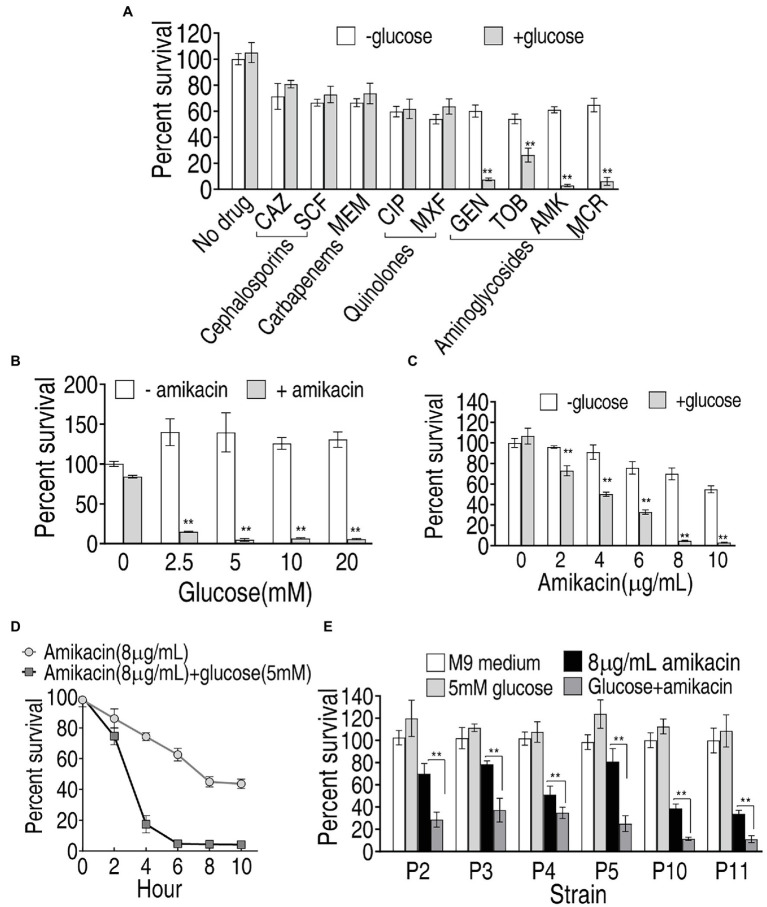
Viability of PA-R16_SCF_ and clinically multidrug-resistant *P. aeruginosa* in the presence of glucose plus antibiotics. **(A)** Percent survival of PA-R16_SCF_ in the presence of the indicated antibiotics. **(B)** Percent survival of PA-R16_SCF_ in the presence of the indicated concentrations of glucose plus amikacin. **(C)** Percent survival of PA-R16_SCF_ in the presence of the indicated concentrations of amikacin plus glucose. **(D)** Percent survival of PA-R16_SCF_ in the indicated incubation periods and in the presence of amikacin plus glucose. **(E)** Percent survival of clinically multidrug-resistant *P. aeruginosa* in the presence of glucose and amikacin. Results are displayed as mean ± SEM and three biological repeats are performed. Significant differences are identified ^**^p < 0.01.

Exogenous glucose elevated intracellular glucose concentration in PA-R_SCF_ and thereby may change PA-R_SCF_ sensitivity to antibiotics since metabolic environment is related to bacterial resistance to drugs (Lee and Collins, 2012; Peng et al., 2015b; Cheng et al., 2018). To screen the best antibiotic that eliminates PA-R_SCF_ in the presence of glucose, percent survival of PA-R16_SCF_ was detected in different classes of antibiotics plus glucose. Exogenous glucose did not promote cephalosporins (SCF; ceftazidime, CAZ)-, carbapenems (meropenem, MEM)-, and quinolones (ciprofloxacin, CIP; moxifloxacin, MXF)-mediated killing but promotes aminoglycosides (Gentamicin, GEN; tobramycin, TOB; amikacin, AMK; micronomicin, MCR) to effectively kill PA-R16_SCF_, where aminoglycosides amikacin showed the best one in the synergistic use with glucose (Figure 6A). Exogenous glucose elevated amikacin-mediated killing in a glucose dose-dependent manner (Figure 6B). The synergistic efficacy with glucose was increased with increasing amikacin dose and incubation period (Figures 6C,D). The combination therapy of glucose with amikacin was also effective to clinically multidrug-resistant *P. aeruginosa* (Figure 6E). These results indicate that the synergistic use of glucose and amikacin is an effective approach to combat lab-evolved and clinical-evolved multidrug-resistant *P. aeruginosa*.

### Mechanisms for Glucose-Potentiated Killing

**Figure 7:**
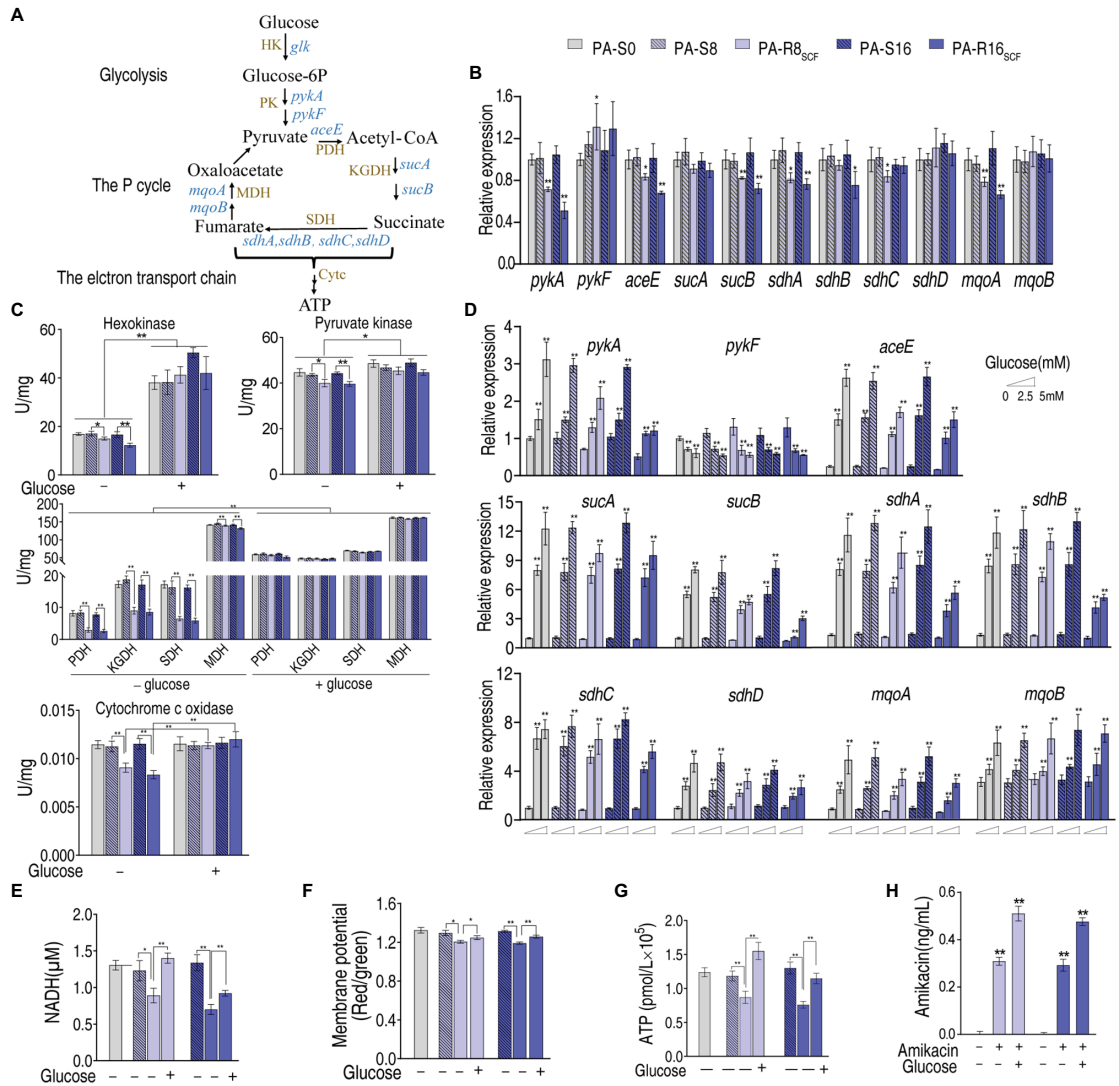
Glycolysis, the P cycle, electron transport chain with and without exogenous glucose in PA-R8_SCF_ and PA-R16_SCF_. **(A)** Metabolic diagram of glycolysis, the P cycle, electron transport chain. **(B)** qRT-PCR for expression of genes encoding glycolysis and the P cycle between PA-R8_SCF_, PA-R16_SCF_, and PA-S8, PA-S16, respectively. **(C)** Activity of enzymes in glycolysis, the P cycle, electron transport chain between PA-R8_SCF_, PA-R16_SCF_, and PA-S8, PA-S16, respectively, in the presence or absence of different concentrations of glucose. **(D)** qRT-PCR for expression of genes encoding glycolysis and the P cycle between PA-R8_SCF_, PA-R16_SCF_, and PA-S8, PA-S16, respectively, in the presence of different concentrations of glucose. **(E–H)** NADH **(E)**, membrane potential **(F)**, ATP **(G)**, and amikacin **(H)** of the two PA-R and three PA-S strains in the presence or absence of glucose. Results are displayed as mean ± SEM, and at least three biological repeats are performed. Significant differences are identified ^*^p < 0.05, ^**^p < 0.01.

To understand how glucose had the potential, we measured expression of key genes and activity of key enzymes in the glycolysis, pyruvate cycle, and electron transport chain because the elevated glucose can flux these metabolic pathways (Figure 7A). Lower expression of these genes was measured between PA-R8_SCF_, PA-R16_SCF_, and PA-S8, PA-S16, respectively (Figure 7B). Consistently, lower activity of these enzymes also characterized between them (Figure 7C). Exogenous glucose promoted the expression of these genes in a dose-dependent manner (Figure 7D). Consistently, activity of these enzymes in the glycolysis, P cycle, and electron transport chain was elevated and similar in PA-R8_SCF_, PA-R16_SCF_ compared with PA-S8, PA-S16, respectively (Figure 7C). NADH, membrane potential, and ATP were decreased in PA-R8_SCF_, PA-R16_SCF_ compared with PA-S8, PA-S16, respectively, which was partly reverted by exogenous glucose (Figures 7E–G). Aminoglycosides uptake is proton motive force-dependent and thereby higher intracellular amikacin was measured in the presence than absence of glucose in PA-R8_SCF_ and PA-R16_SCF_ (Figure 7H). These results suggest that exogenous glucose fluxes to glycolysis, the P cycle, and electron transport chain, which promotes amikacin uptake and leads to the high killing efficacy.

## Discussion

Antibiotic resistance crisis is happening due to the drying up of the antibiotic discovery pipeline, which results in unchecked spread of antibiotic-resistant pathogens (Lewis, 2020). Understanding for antibiotic resistance mechanisms and development of novel control approach are especially important. Based on the findings that the glucose and its downstream metabolic pathways are depressed in lab-evolved SCF-resistant and clinically isolated multidrug-resistant *P. aeruginosa*, the present study explores why glucose is reduced in these bacterial strains and which antibiotic is potentiated by exogenous glucose to achieve the best killing efficacy to these strains.

*Pseudomonas aeruginosa* is a metabolically versatile bacterium that arises many of key adaptations, which are centered on core metabolism, during infection (Dolan, 2020). Recent metabolomic studies of *P. aeruginosa* have provided valuable information about metabolic pathways, leading to an understanding of the adaptations of bacterial strains to a host environment (Chen et al., 2017; Han et al., 2018; Mielko et al., 2019; Allobawi et al., 2020). Moreover, the metabolomics studies have resulted in new drug development and/or elaboration of new treatment strategies for *P. aeruginasa*. Meylan et al. (2017) show that fumarate potentiates tobramycin susceptibility, while glyoxylate promotes tolerance. Kuang et al. (2021) demonstrate that exogenous fumarate, NADH, nitrate, and nitrite promote NO level and thereby potentiate SCF-mediated killing. Glucose as a central carbohydrate is metabolized during the early growth in *P. aeruginosa* (Frimmersdorf et al., 2010). Depressed glucose and inactivated central carbon metabolism have been recognized in antibiotic-resistant bacteria, although mechanisms are unknown (Peng et al., 2015b; Zhang et al., 2019). However, information regarding the role of glucose is not available in antibiotic-resistant *P. aeruginosa*. The present study shows that lower expression of genes encoding glucose uptake and phosphorylation in PA-R_SCF_ than PA-S, indicating that the decreased uptake and phosphorylation contribute to the depressed glucose. This finding is supported by the event that the depressed glucose is linked to the reduced glycolysis, P cycle, and electron transport chain, while exogenous glucose activates these metabolic pathways of the central carbon metabolism in PA-R_SCF_. These results not only reveal that the depressed glucose is attributed to the limited glucose uptake, but also provide a previously unknown target to combat antibiotic-resistant bacteria.

Alternative approaches emerge as the times demand (Bechinger and Gorr, 2017; Gordillo Altamirano and Barr, 2019; Jiang et al., 2020b; Yang et al., 2021), where metabolome-reprogramming is a recently developed approach to restore the conventional antibiotics-mediated killing efficacy (Peng et al., 2015a,b). In the reported metabolome-reprogramming approach, crucial biomarkers from comparison between antibiotic-sensitive and -resistant metabolomes are identified and then are used to potentiate the antibiotics to effectively kill the antibiotic-resistant bacteria. Here, SCF-sensitive and -resistant metabolomes were compared and glucose was identified as the most crucial biomarker. However, exogenous glucose did not promote SCF-mediated killing, instead it potentiated amikacin of aminoglycosides to eliminate lab-evolved PA-R and clinically isolated multidrug-resistant *P. aeruginosa*. Importantly, out of four classes of antibiotics tested, cephalospotines, carbapenems, quinolones, and aminoglycosides, only four types of aminoglycosides showed the elevated killing. Therefore, widespread screen of antibiotics is required for identification of the most effective drug in the synergistic use with the crucial biomarker. More importantly, both SCF-resistant and amikacin-resistant *P. aeruginosa* is a big challenge to uncontrolled infection in clinic (Huai et al., 2019). Amikacin is one of antibiotics for the treatment of *P. aeruginosa* with difficult-to-treat resistance infections (Tamma et al., 2021). The glucose-potentiated amikacin-mediated killing provides a potential approach to eliminate these antibiotic-resistant pathogens. The finding deepens the understanding on metabolome-reprogramming to potentiate antibiotic-mediated killing and expands the application on selecting antibiotics to achieve the best killing efficacy.

Additionally, the present study reveals that the depressed glucose due to decreased uptake leads to the inactivation of glycolysis, the P cycle, and electron transport chain, where the P cycle bridges glycolysis to electron transport chain directly (Su et al., 2018). The finding on the P cycle is originated from *E. tarda* and *E. coli* based on antibacterial efficacy. Oxaloacetate-phosphoenolpyruvate-pyruvate-AcCoA-citrate, oxaloacetate-pyruvate-AcCoA-citrate, and malate-pyruvate-AcCoA-citrate are three ways by which the TCA cycle is connected to the P cycle, Beside the two bacteria, there is a proof-of-principle for *V. anguillarum*, *V. alginolyticus*, *V. parahaemolyticus*, *V. vulnificus*, *V. fluvialis*, and *Photobacterium damsel* (Su et al., 2018). However, *P. aeruginosa* is not involved. Recently, Kohlstedt and Wittmann develop a GC–MS-based ^13^C metabolic flux analysis to resolve the parallel and cyclic glucose metabolism of *Pseudomonas putida* KT2440 and *P. aeruginosa* PAO1. They have shown that oxaloacetate-phosphoenolpyruvate-pyruvate-AcCoA-citrate and malate-pyruvate-AcCoA-citrate occur, but the major flux is from phosphoenolpyruvate to pyruvate to AcCoA, with appreciable amount of carbon going to oxalacetate from pyruvate (Kohlstedt and Wittmann, 2019), which is consistent with oxaloacetate instead of AcCoA as a fuel in the P cycle (Su et al., 2018). On the other hand, GC–MS, NMR, and UPLC-MS are three main analytical technologies supporting metabolomics with their own advantages and disadvantages (Peng et al., 2015a; Salzer and Witting, 2021). In our case, the reduced glycolysis, pyruvate cycle and electron transport chain was identified by GC–MS and then validated by qRT-PCR or/and enzyme measurement, which is further supported by NADH, PMF and ATP. In addition, the depressed glucose was also demonstrated by commercial kit. Therefore, the results on the reduced glycolysis, pyruvate cycle, and electron transport chain caused by the depressed glucose due to decreased uptake are guaranteed in the present study.

*P. aeruginosa* has its striking capacity for antibiotic resistance development. Molecular mechanisms of the antibiotic resistance include reduced influx, increased efflux, prevention of access to target, changes in antibiotic targets by mutation, modification, and protection of targets, and direct modification of antibiotics (Jeukens et al., 2019; Peng et al., 2019). The present study shows that exogenous glucose elevates intracellular amikacin by 1.63-fold, indicating that the synergistic use of glucose and amikacin can effectively kill antibiotic-resistant *P. aeruginosa via* promotion of antibiotic uptake. Therefore, the reverting of the reduced influx by crucial biomarkers provides a useful approach to eliminate the difficult-treatment pathogens.

However, the present study is carried out only *in vitro*. Therefore, further study *in vivo* in animal models of infection is needed to validate this approach and its utility for treating antibiotic-resistant infections in the future.

## Conclusion

**Figure 8:**
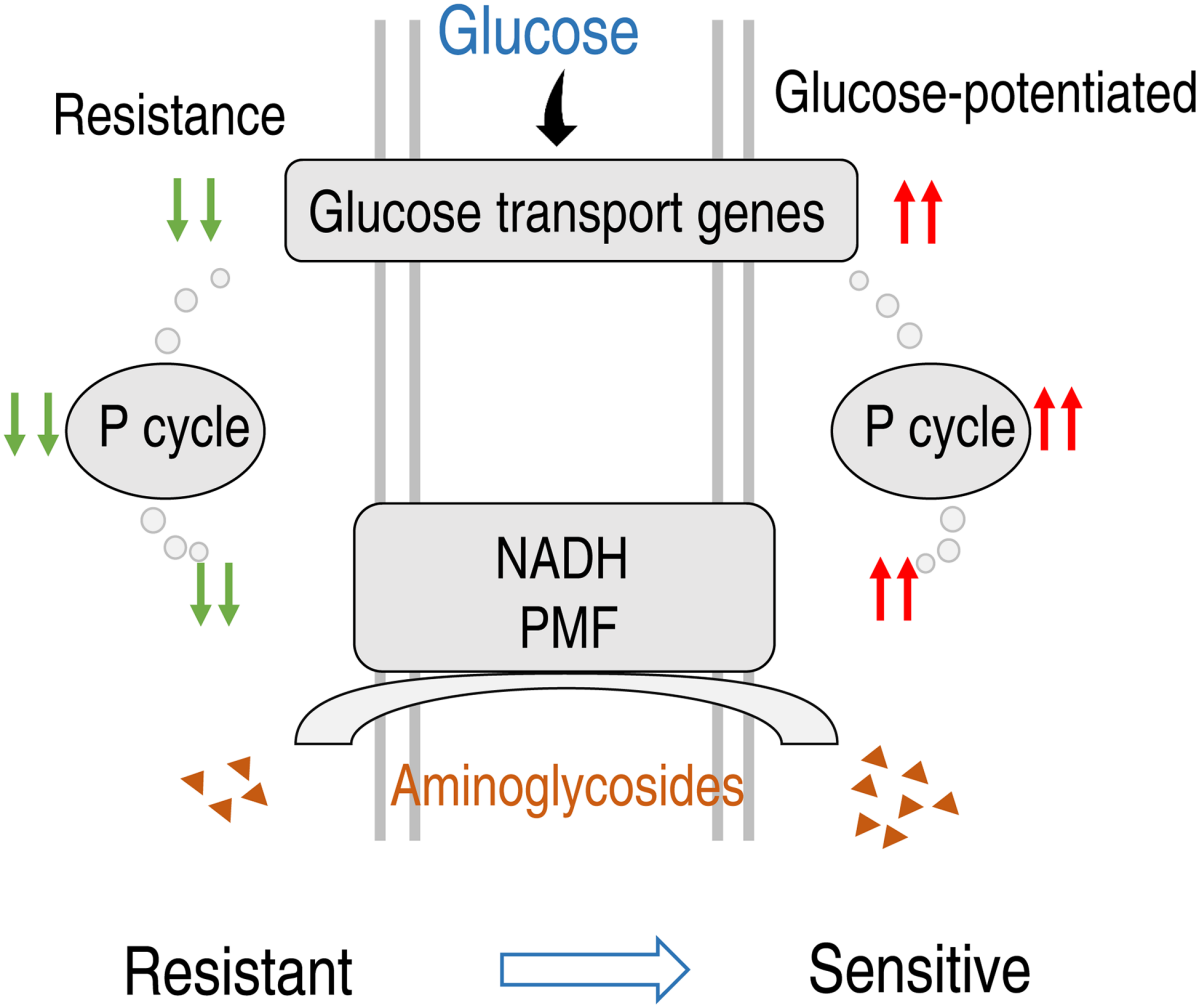
Diagram for glucose-mediated SCF-resistant mechanism and reverting by exogenous glucose.

In summary, the present study shows that PA-R_SCF_ strains have different ability in utilization of sugars from PA-S strains and thereby metabolic profiles are shifted. The shifted metabolomes characterize the reduced glycolysis and the P cycle of the central carbon metabolism and depressed glucose as the key metabolic pathways and the most crucial biomarker. The depressed glucose is related to the downregulated genes working for glucose transport. Exogenous glucose activates expression of these genes and elevates intracellular glucose leading to activation of glycolysis, the P cycle, and electron transport chain. These activated metabolic pathways cause the elevation of membrane potential and in turn result in antibiotic uptake and thereby effectively eliminate lab-evolved PA-R and clinically isolated multidrug-resistant *P. aeruginosa* (Figure 8). Therefore, the present study leads to two major findings: (1) The depression of glucose uptake is responsible for the reduction of intracellular glucose and (2) amikacin is the best antibiotic potentiated by glucose to eliminate multidrug-resistant *P. aeruginosa*.

## Data Availability Statement

The original contributions presented in the study are included in the article/Supplementary Material, further inquiries can be directed to the corresponding author.

## Author Contributions

Z-gC conceptualized, designed the project, and wrote the manuscript. Z-gC and T-tZ interpreted the data. X-kT, Y-bS, H-qY, Z-yD, HY, and K-xY performed the data analysis and the experiments. All authors contributed to the article and approved the submitted version.

## Funding

This work was financially supported by grants from Guangdong Basic and Applied Basic Research Foundation (2021A1515010133 and 2019A1515011441).

## Conflict of Interest

The authors declare that the research was conducted in the absence of any commercial or financial relationships that could be construed as a potential conflict of interest.

## Publisher’s Note

All claims expressed in this article are solely those of the authors and do not necessarily represent those of their affiliated organizations, or those of the publisher, the editors and the reviewers. Any product that may be evaluated in this article, or claim that may be made by its manufacturer, is not guaranteed or endorsed by the publisher.

## Supplementary Material

The Supplementary Material for this article can be found online at: https://www.frontiersin.org/articles/10.3389/fmicb.2021.800442/full#supplementary-material

Supplementary Figure 1 Pathway enrichment. **(A)** Pathway enrichment of differential metabolites in PA-R8_SCF_ and PA-R16_SCF_ based on controls PA-S8 and PA-S16, respectively. Significant enriched pathways are selected to plot. Value of *p* < 0.05. **(B)** Integrative analysis of metabolites in significantly enriched pathways. Yellow color and blue color indicate increased and decreased metabolites, respectively.

## Footnotes

^1^ http://cran.r-project.org/web/packages/gplots/

^2^ http://www.kegg.jp/

^3^ https://pubchem.ncbi.nlm.nih.gov/

^4^ http://www.genome.jp/kegg

^5^ http://metpa.metabolomics.ca
