# Supplementary figures and images for: Glucose-Potentiated Amikacin Killing of Cefoperazone/Sulbactam Resistant Pseudomonas aeruginosa
Source: Front Microbiol. 2022 Mar 3;12:800442. doi: 10.3389/fmicb.2021.800442 (PMC8928219; doi:10.3389/fmicb.2021.800442)

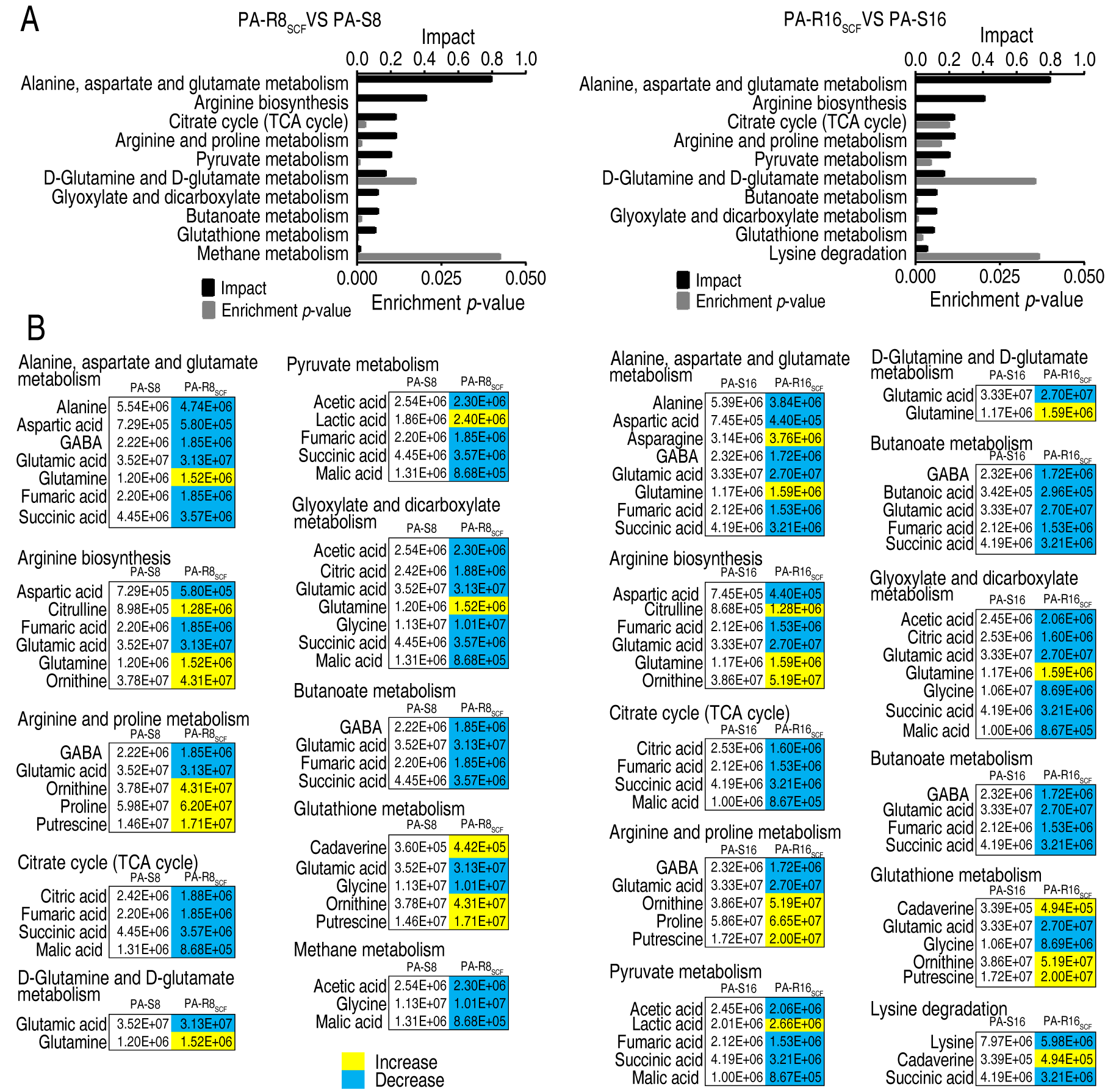

Supplement: Supplementary Figure 1 — Pathway enrichment. (A) Pathway enrichment of differential metabolites in PA-R8SCF and PA-R16SCF based on controls PA-S8 and PA-S16, respectively. Significant enriched pathways are selected to plot. Value of p < 0.05. (B) Integrative analysis of metabolites in significantly enriched pathways. Yellow color and blue color indicate increased and decreased metabolites, respectively. [file Image_1.TIF]
