# Supplementary material for: Glucose-Potentiated Amikacin Killing of Cefoperazone/Sulbactam Resistant Pseudomonas aeruginosa
Source: Front Microbiol. 2022 Mar 3;12:800442. doi: 10.3389/fmicb.2021.800442 (PMC8928219; doi:10.3389/fmicb.2021.800442)
Supplement: Supplementary file 2 [file Table_1.DOCX]

Table 1 Primers for qRT-PCR

| Gene |  | Primer (5'-3') |  | Gene |  | Primer (5'-3') |
| --- | --- | --- | --- | --- | --- | --- |
| 16S rRNA | Forward | CAAAACTACTGAGCTAGAGTACG |  | *eda* | Forward | CAGCGAGCGATAACCGTACT |
|  | Reverse | TAAGATCTCAAGGATCCCAACGGCT |  |  | Reverse | CTGATCCACACCGAGCAGTT |
| *pykA* | Forward | AAGTTCCGCTTCTCCACCAG |  | *gad* | Forward | TGCATACCAACCCCTTCGTC |
|  | Reverse | CTTGATGTCGGCCTTGTCCT |  |  | Reverse | CGAGGTCGAGGTAGTTGTCG |
| *pykF* | Forward | TTCGCCATCATCTCTACCGC |  | *kguK* | Forward | GGGTTCCAGATGAAGTCCCG |
|  | Reverse | GCATCCAGAAACGCATCGTC |  |  | Reverse | TCAGTTCGTGGGACAGTTCG |
| *aceE* | Forward | CAGTTCCCCACCGTATCCA |  | *gcd* | Forward | GCGTACTGCTCTTCGTCGTA |
|  | Reverse | CGAAGATCAGGTTGTCGAGTTT |  |  | Reverse | GCTCGAACTGACCAACGAGA |
| *sucA* | Forward | CGCCAAGCAGCGTACTACCC |  | *oprB* | Forward | AGGTTCTGGAAATCGCAGGG |
|  | Reverse | GGCGAAGCCCCAGTTGA |  |  | Reverse | CGAGTTCCAGTTCACCGTCA |
| *sucB* | Forward | GTGGCGTGTTCGGTTCCCT |  | *oprB1* | Forward | TGACGGTGAACTGGAACTCG |
|  | Reverse | ATCAGACGGTGGTCGTAGGA |  |  | Reverse | GCCGAACTCGAAATGGATGC |
| *sdhA* | Forward | GAAGAAGTCGCTCCGCTCAA |  | *glk* | Forward | TAATGTAGACGCCACCGAGC |
|  | Reverse | GAATGCCTGGCTCTTGTCG |  |  | Reverse | CCTGCTCGCCCTCTATGAAA |
| *sdhB* | Forward | CAAGGAACAGGACGAGGGC |  | *gap* | Forward | GCCTACACCAACGACCAGAA |
|  | Reverse | AGAAGGACGGGCAGGAGGT |  |  | Reverse | TGACCTCGTCTACGCTGGTA |
| *sdhC* | Forward | CCGTGAATAGCAAACGACCTG |  | *kguT* | Forward | AGCCAGTGAAGAACTACCGC |
|  | Reverse | GGCAATACCGAGGAACAGGA |  |  | Reverse | GGCGTAGGAACCGTAGAAGG |
| *sdhD* | Forward | TTCCTGCTGGGCTACCTCATT |  | *gltK* | Forward | TACAGGGCGTAGGACTGGAA |
|  | Reverse | CAGGGTCAACAGGCTGAAGAT |  |  | Reverse | GAAGATCGACTCCGGCGAAT |
| *mqoA* | Forward | CCAATGGCGACAAGGAAAC |  | *gntP* | Forward | TGATCCCGCTGGTGTTCATC |
|  | Reverse | CGGACCGAACAGCAGAACC |  |  | Reverse | GGCCGTAGAGGATGGTCTTG |
| *mqoB* | Forward | CCCCTATGCCGGTTTCTCC |  | *zwf* | Forward | GGACCTTGACCTTCTCGTCG |
|  | Reverse | CGGGTCAGGTCCATGTTGT |  |  | Reverse | CGGATCGACCATTACCTGGG |
